# Comparison of Ponseti and Kite’s method of treatment for idiopathic clubfoot

**DOI:** 10.4103/0019-5413.77143

**Published:** 2011

**Authors:** Ashok K Shyam

**Affiliations:** *Sancheti Institute for Orthopaedics and Rehabilitation, Shivaji Nagar, Pune, Indian Orthopaedic Research Group, Thane, Maharashtra, India E-mail: drashokshyam@yahoo.co.uk*

Sir,

I have with interest read the article by Rijal **et al**. entitled “Comparison of Ponseti and Kite’s method of treatment for idiopathic clubfoot” recently published in Indian Journal of Orthopaedics.^1^

Following are my concerns regarding the article:

1. The Ponseti method is currently the most accepted method of conservatively treating the idiopathic clubfeet. Kites method was a rebending the bend to straighten the curve while Ponseti’s method is based on well-studied pathoanatomy. As mentioned by the authors themselves, the success rate of Kite is maximum 58% while that of Ponseti is 78–98%.^2^–^4^ The Kite’s method has been shown to be lengthy and 50–75% needed soft tissue release.^5^ Reviews on the subject now do not even take into account Kite’s method as an acceptable treatment for clubfoot.^6^,^7^ With this background, I wish to enquire the need and ethics of subjecting 30 feet’s in children to Kite’s method. Is there really a need for doing such a randomized trial?
2. The authors quote that they could find only one randomized trial comparing the Ponseti with Kite’s method;^8^ however, this study was published in 2008 while the authors have begun their study in 2005. Only studies available before the time of starting the study can justify the rationale of the study. Since the clinical use of ponseti technique is well established, there seems no justification to subject children to Kite’s manipulation for the sake of comparing a scoring system or quantifying time duration for correction.
3. The authors claim this to be a randomized controlled trial, which according to the IJO policy should be according to the CONSORT guidelines. I find few things missing:
  - No mention of sample size calculation
  - The method of allocation concealment used
  - No mention of whether a well-informed consent was taken from the parents of the children randomized for the study
  - If any losses to follow-up, drop-outs, or failure of treatment, etc.
  - No mention of the end-points of the study
4. The results have been analyzed in subgroups. However according to me, the study is using two different kinds of designs, a randomized control trial to allocate the intervention and matched pair designs to analyze the results. This in itself is conflicting. If we have randomized correctly, there is no need for any such subgroup-matched pair analysis, which will drastically increase the alpha error.
5. The authors mention that “The sample size is adequate as even in subgroups the differences have attained statistical significance.” Such posthoc assumptions on sample size in a RCT cannot be done and has no meaning.
6. Presentation of standard error and confidence interval statistics rather than P value would have been more informative. The best would have been to plan this study as a randomized controlled superiority trial supporting Ponseti method over Kite’s method with predefined end-points and minimal clinically significant difference in the Pirani score.
